# Nevus lipomatosus superficialis mimicking ectopic breast tissue: A rare case report

**DOI:** 10.1097/MD.0000000000041339

**Published:** 2025-02-07

**Authors:** Hui Beom Hwang, Sehoon Yoon, Ki Yong Hong

**Affiliations:** a Department of Plastic and Reconstructive Surgery, Seoul National University Hospital, Seoul National University College of Medicine, Seoul, Korea.

**Keywords:** connective tissue diseases, lipoma, skin neoplasms

## Abstract

**Rationale:**

Diagnosis of solitary superficial lipomatous mass may vary depending on circumstances. Although preliminary diagnosis can be made through clinical symptoms and imaging studies, final diagnosis can change through histological examination. This is the first reported case of nevus lipomatosus superficialis (NLS) resembling ectopic breast tissue which may provide valuable insights for clinicians managing similar lesions.

**Patient concerns:**

A 41-year-old female had a solitary mass on her left lower flank, lasting more than 10 years with cyclic pain corresponding to her menstrual cycle.

**Diagnoses:**

A computed tomography scan suggested benign subcutaneous lipoma, but histopathological examination after surgery confirmed the diagnosis of NLS.

**Interventions:**

Surgical excision was performed without complications.

**Outcomes:**

No regrowth of the lipomatous mass was observed at the 6-month follow-up, and there was no recurrence of cyclic pain.

**Lessons:**

This report aims to discuss an unusual presentation of NLS that mimics ectopic breast tissue and causes cyclic pain related to the menstrual cycle. Understanding this case provides insights into the potential hormonal influence on focal adipose tumors and the challenges in differential diagnosis.

## 1. Introduction

Nevus lipomatosus superficialis (NLS), first reported by Hoffman and Zurhelle in 1921, is a rare benign hamartomatous skin lesion characterized by the ectopic dermal deposition of mature adipose tissue.^[1]^ NLS is classified into 2 forms: the classical form, which presents as multiple soft, pedunculated papules or nodules typically located in the pelvic girdle area, and the solitary form, which manifests as a dome shaped single sessile papule or nodule with no specific site predilection.^[2,3]^ While the lesion is usually asymptomatic, symptoms such as pain, ulceration, or foul odor can occasionally occur.^[4,5]^ As NLS can be clinically challenging to distinguish from other lesions, a definitive diagnosis is made through histological examination.

NLS has been reported to occur on almost all body parts, including the face, scalp, shoulder, lower back, pelvic girdle, and lower extremity.^[2,6–8]^ In most cases, however, the lesion is asymptomatic, with only a few cases presenting with pain or ulceration.^[4,5]^ Here, we present an unusual case of a patient who suffered from this rare skin lesion with cyclic pain associated with her menstrual cycle.

## 2. Case

According to the policies of our research institution, Institutional Review Board (IRB) approval is not required for case reports involving a single individual who cannot be identified. Therefore, IRB approval was waived for this case report. Written informed consent was obtained from the patient.

**Figure 1.**
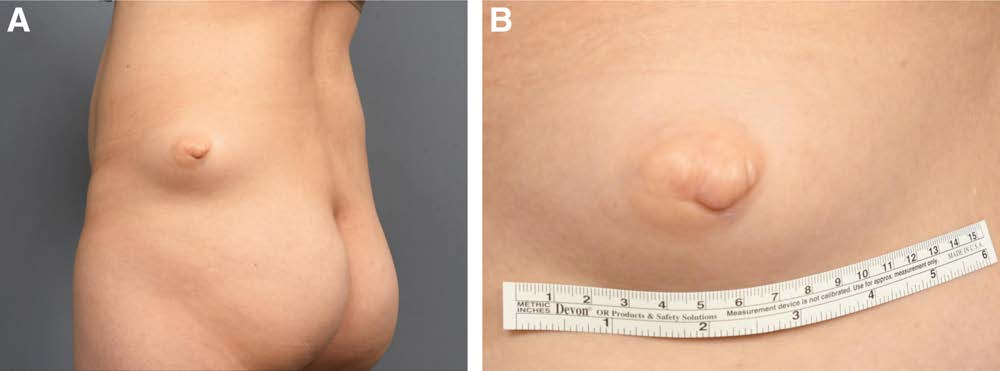
Initial medical photograph of the lesion. (A) A solitary lesion on the left flank with a centrally elevated, pigmented, rounded nodule. (B) A close-up image of the lesion showing a 7.5 × 6 cm mass.

A 41-year-old female patient visited the clinic with a solitary mass on her left lower flank. She first noticed the lesion about 10 years ago, and the mass had gradually increased in size (Fig. 1). Additionally, as the mass grew larger, the lesion recently began to present with cyclic pain associated with her menstrual cycle. The patient reported no family history of similar lesions.

**Figure 2.**
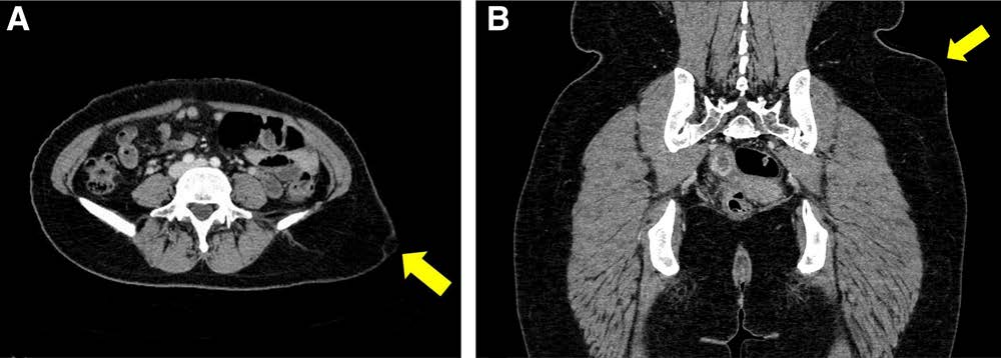
Computed tomography scan revealing a 5.7 × 8.4 × 5.8 cm fatty mass (yellow arrow) in the subcutaneous layer of the left lower flank. (A) Cross-sectional view and (B) coronal view.

Physical examination revealed a 7.5 × 6 cm solitary, skin-colored, immobile nodule with an elevated center on the surface. Abnormal skin changes such as ulceration, café-au-lait macules or hypertrichosis were not present. Due to pain’s correlation with the menstrual cycle, ectopic breast tissue was suspected. However, computed tomography (CT) scan, showed a 5.7 × 8.4 × 5.8 cm fatty mass in the subcutaneous layer of the left lower flank suggesting a benign subcutaneous lipoma (Fig. 2).

During the work-up, a CT scan revealed multiple myomas measuring up to 6 cm, along with an enlarged uterus. The patient subsequently underwent a total laparoscopic hysterectomy. However, after the gynecologic surgery, the cyclic pain in the left lower flank slightly improved but still persisted.

**Figure 3.**
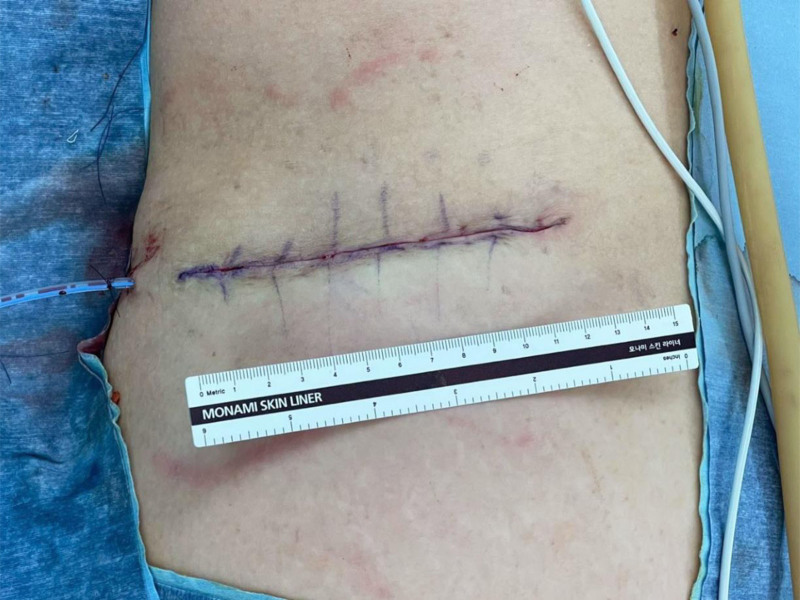
Postoperative medical photograph showing the mass removed from the subcutaneous layer and the wound closed in layers.

**Figure 4.**
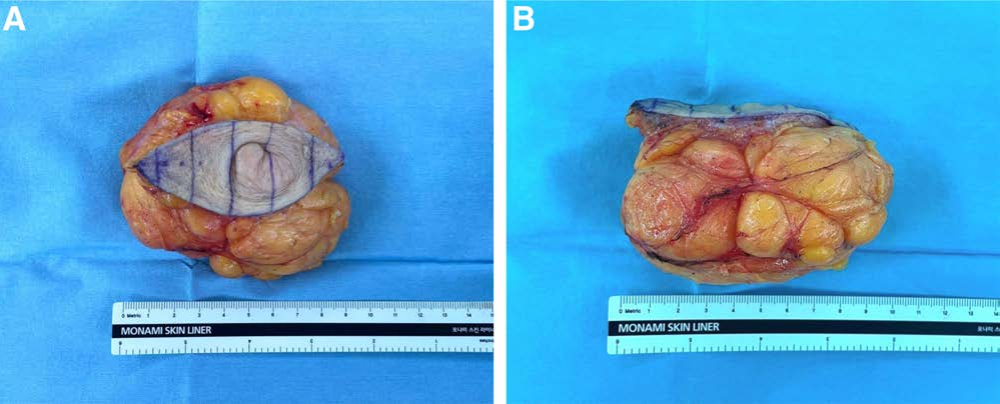
The specimen was sent for histopathological examination. (A) An 8.7 × 6.0 × 6.0 cm mass, including the attached skin, was excised. (B) The specimen consists of yellowish, soft fatty tissue.

**Figure 5.**
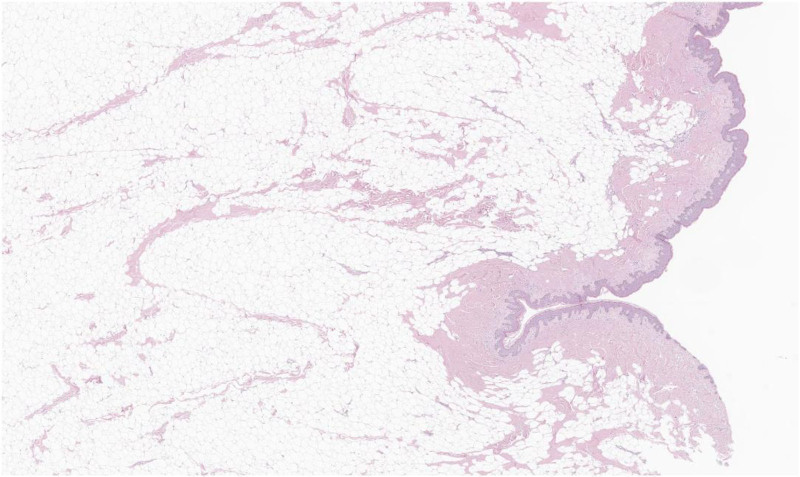
Histological image demonstrating an aggregation of mature adipocytes in the reticular dermis under the microscope in ×40 magnification. No glandular tissue was observed.

**Figure 6.**
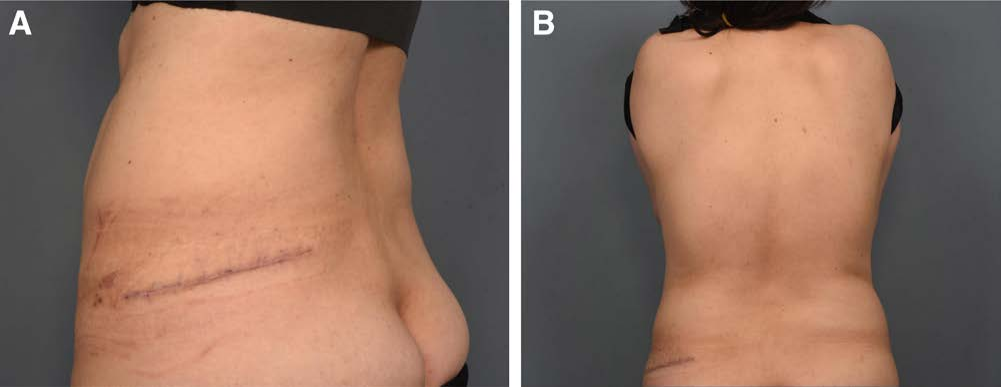
Medical photograph taken 1 mo after surgical excision. No recurrence was observed at the 6-mo follow-up. (A) Lateral view and (B) posterior view.

The patient requested surgical treatment due to pain and cosmetic reasons. An elliptical incision was made around the mass, followed by subcutaneous dissection. A soft, lobulated, and encapsulated lipomatous mass was removed. The wound was closed in layers (Fig. 3). An 8.7 × 6.0 × 6.0 cm mass composed of yellowish, soft fatty tissue was sent for histopathological examination (Fig. 4). Microscopy revealed an aggregation of mature adipocytes throughout the reticular dermis, embedded among the collagen bundles, constituting more than 50% of the dermis. Glandular tissue was not found upon microscopy, confirming the absence of ectopic breast tissue. These findings established the final diagnosis as the solitary form of NLS (Fig. 5). The patient was discharged without any complications, and follow-ups were conducted at 1 month and 6 months. A medical photo taken at the 1-month follow-up showed no signs of recurrence. At the 6-month follow-up, the patient remained symptom-free, with no evidence of lesion regrowth or recurrence of cyclic pain. (Fig. 6).

## 3. Discussion

Nevus lipomatosus superficialis is an uncommon idiopathic hamartomatous skin anomaly with various presentations. The classical form of NLS typically arises congenitally or during the first 2 to 3 decades of life and is predominantly located on the lower back, buttocks, and thighs.^[2,3]^ In contrast, the solitary form generally appears later, typically between the third and sixth decades of life, and can occur on various body sites, including the face and genitalia.^[2,9–11]^ In our case, the lesion is consistent with the solitary form.

Several theories have been proposed, but the pathogenesis of NLS is still not clear. Hoffman and Zurhelle argue that fat deposition in the dermis arises from degenerative changes in the connective tissue.^[1]^ Other theories consider it to be a true nevus resulting from the focal heterotopic development of adipose tissue.^[12]^ Additionally, some suggest that adipocytes originate from pericytes of dermal blood vessels.^[2]^ The possibility of pathogenesis due to a chromosomal abnormality (2p24 deletion) has also been reported.^[13]^

NLS is usually reported as asymptomatic. Although there are a few cases with accompanying symptoms such as pain or ulceration, to our knowledge, this is the first report of NLS presenting with cyclic pain dependent on the menstrual cycle.^[4,5]^ In our case, the lesion was in a solitary form, and the protruding central area somewhat resembled a nipple. In terms of the location, ectopic breast tissue typically develops along the mammary ridge. However, it can rarely appear outside the mammary ridge, such as on the face, foot, and vulva.^[14–16]^ These unusual presentations are thought to develop from modified apocrine sweat glands.^[17]^ Therefore, based on the morphological features of the lesion and the patient’s symptoms, it could have been misdiagnosed as ectopic breast tissue.

In our case, the patient was diagnosed with multiple myomas measuring up to 6 cm. Given the large size and number of myomas, they could have contributed to the cyclic pain observed in the NLS of the left flank. However, since the cyclic pain in the NLS persisted even after the total laparoscopic hysterectomy, it is more likely that the pain is directly associated with the NLS lesion itself rather than the myomas.

NLS should be clinically differentiated from other mass-like lesions. Representative conditions requiring differential diagnosis include papilloma, acrochordon, fibrolipoma, neurofibroma, and lymphangioma.^[2,18]^ However, our case emphasizes the importance of considering other mass-like lesions that may cause cyclic pain, such as endometriosis within a subcutaneous lesion or fibrocystic disease. Histopathological examination is essential for a definitive diagnosis, with the presence of mature adipocytes in the reticular dermis, which may also extend into the papillary dermis.^[9]^

Since there have been no reports of malignant transformation, treatment is typically carried out for cosmetic purposes or to alleviate the patient’s symptoms.^[2]^ Surgical excision is the treatment of choice, and recurrence is rare. As alternatives, nonsurgical treatments, including CO_2_ laser, intralesional phosphatidylcholine and sodium deoxycholate injections, and cryotherapy, may also be considered.^[19,20]^ In our case, the patient opted for surgical excision due to pain and cosmetic concerns.

There has been little research on the association between this lesion and hormonal effects during the menstrual cycle. In 1 case, pregnancy-onset NLS was reported over the nipple.^[21]^ A 29-year-old female was diagnosed with NLS at 26 weeks of gestation. The lesion was asymptomatic and slowly growing over the right nipple. The authors suggested that physiological skin changes during pregnancy or undocumented hormonal influences might induce aberrant adipocyte hyperplasia. It is well known that ovarian hormones regulate lipolysis and lipid storage in adipose tissue.^[22–24]^ Although there is controversy over the role of estrogen receptors in fat accumulation, numerous studies support that estrogen promotes the accumulation of subcutaneous adipose tissue in the gluteal and femoral regions, especially in premenopausal women.^[25]^ Therefore, in our case, changes in ovarian hormones during the menstrual cycle might have caused adipose tissue expansion via adipogenesis or hypertrophy of adipocytes. This can lead to expansion of the lipomatous mass, potentially provoking cyclic pain associated with the menstrual cycle. This mechanism differs from the 1 causing mastalgia in ectopic breast tissue, where hormones directly affect the mammary gland tissue periodically.

This study has several limitations. First, as a single-case report, the findings are inherently limited in their generalizability and may not represent broader patterns of NLS presentations. Second, while the relationship between ovarian hormones and the expansion of the lipomatous mass was hypothesized, the precise mechanism remains unclear, necessitating further research. To understand the precise mechanism of hormone induced pain from lipomatous mass, further research is needed on the relationship between ovarian hormones and focal adipose tumors.

## Author contributions

**Conceptualization:** Ki Yong Hong.

**Supervision:** Ki Yong Hong.

**Writing – original draft:** Hui Beom Hwang, Sehoon Yoon.

**Writing – review & editing:** Hui Beom Hwang, Sehoon Yoon, Ki Yong Hong.
